# Platelets-Derived miR-200a-3p Modulate the Expression of ET-1 and VEGFA in Endothelial Cells by Targeting MAPK14

**DOI:** 10.3389/fphys.2022.893102

**Published:** 2022-06-09

**Authors:** Jie Yang, Hong Xu, Kejie Chen, Danni Zheng, Shuang Liu, Xia Zhou, Yapeng Lin, Hang Cheng, Qin Luo, Min Yang, Xiaoyan Yan, Junli Hao

**Affiliations:** ^1^ Department of Neurology, Sichuan Provincial People’s Hospital, University of Electronic Science and Technology of China, Chengdu, China; ^2^ Department of Neurology, The First Affiliated Hospital of Chengdu Medical College, Chengdu, China; ^3^ School of Pharmacy, Chengdu Medical College, Chengdu, China; ^4^ School of Public Health, Chengdu Medical College, Chengdu, China; ^5^ Biomedical Informatics and Digital Health, School of Medical Sciences, University of Sydney, Sydney, NSW, Australia; ^6^ School of Biomedical Sciences and Technology, Chengdu Medical College, Chengdu, China

**Keywords:** acute ischemic stroke, platelets, vascular endothelial cells, miRNA, MAPK14

## Abstract

The interaction between platelets and vascular endothelial cells plays a pivotal role in the pathophysiology of acute ischemic stroke (AIS), especially in atherosclerosis formation. However, the underlying mechanism is not entirely clear. The aim of this study was to elucidate the role of platelets-derived miRNA in the development of atherosclerosis and AIS. We evaluated the miRNA expression profiles of serum microvesicles (MV) in five AIS patients and five healthy controls using RNA-seq, and then measured the levels of selected platelets derived miRNAs by qRT-PCR. miR-200a-3p expression in the serum MV and platelets had increased to 1.41 (*p* < 0.05) and 3.29 times (*p* < 0.001), respectively, in AIS patients compared with healthy controls, and was modified by severity of AIS. We transferred Cy5-miR-200a-3p into platelets, collected and identified platelets-derived MV (PMVs). Then, the gene expression of p38 MAPK/c-Jun pathway was analyzed using both miR-200a-3p gain- and loss-of-function experiments and PMVs coincubation with HUVEC. The results showed that activated platelets remotely modulated endothelins 1 (ET-1) and vascular endothelial growth factor A (VEGFA) levels in HUVEC through the release of miR-200a-3p-containing PMVs *via* targeting MAPK14. The results of ROC analyses showed that combination of platelet miR-200a-3p, serum ET-1 and VEGFA levels had an AUC of 0.817, a sensitivity of 70%, and a specificity of 89%. Our results presented new evidence that activated platelets could remotely modulate ET-1 and VEGFA expression in HUVEC via releasing miR-200a-3p-enriched PMVs, which provides a potential miRNA-based predictive biomarker and therapeutic strategy for atherosclerosis and AIS.

## Introduction

Acute ischemic stroke (AIS) is the one of the leading causes of mortality and disability throughout the world (Akella et al., 2019; Herpich and Rincon, 2020). There are five different AIS subtypes according to the TOAST classification: large artery atherosclerosis (LAA), cardioembolic infarct (CE), lacunar infarct (LAC), stroke of other determined etiology (ODE), and stroke of undetermined etiology (UDE). LAA accounts for about 45% of all strokes, and a significant contributor to stroke-related deaths (Maida et al., 2020). The typical pathology of atherosclerosis is atheroma formation, which contains a large number of necrotic cells and inflammatory cells (Luo et al., 2017). In the vicinity of the lesion, platelets are further activated under the stimulation of subendothelial factors. In turn, activated platelets, interacting with vascular endothelial cells as well as other circulating immune cells in inflammatory condition, play an important role in the development of endothelial dysfunction and atherosclerosis (Lievens and von Hundelshausen, 2011; Pan et al., 2014).

miRNAs are about 22 nucleotide long non-coding RNAs and highly conserved. They regulate mRNA degradation or translational suppression by binding to complementary sequences in 3′ untranslated region (3′UTR) or open reading frame of target mRNAs (Bijak et al., 2016; Li et al., 2016), and they play a key role in modulating various biological processes and serve as effective biomarkers for diagnosis and prognosis of various diseases (Bijak et al., 2016; Dovizio et al., 2018; Alexandru et al., 2021). Recent studies have demonstrated that anucleate platelets also contain abundant miRNAs and miRNA processing mechanisms, such as Dicer, RNA-binding protein 2, and AGO2, that convert pre-miRNA into mature miRNA (Laffont et al., 2013; Rowley et al., 2016). Platelets-derived microvesicles (PMVs) could deliver miRNAs to endothelial cells to affect its function. Gidlof et al. and Li et al. have revealed that functional miRNAs released from activated platelets, could enter endothelial cells and regulate intercellular adhesion molecule 1 (ICAM) expression (Gidlöf et al., 2013; Li et al., 2017). Yi et al. have demonstrated that PMVs could deliver miR-223 into human umbilical vein endothelial cells (HUVEC) and promote apoptosis by targeting endothelial insulin-like growth factor 1 receptor (Pan et al., 2014).

These findings indicate that miRNAs secreted from platelets *via* PMVs could cause endothelial cell dysfunction and contribute to the atherothrombosis and AIS. However, little is known about the types and functions of platelets derived miRNA in AIS. Therefore, to investigate the role of new miRNAs in the AIS, we detected the miRNA expression profile in the serum MV from AIS patients and healthy subjects by RNA-seq, examined the miRNA expression in the platelets and we further explored the underlying mechanism of differentially expressed miRNA involved in the AIS development.

## Materials and Methods

### Participant Recruitment

For miRNA profile expression analysis, 53 AIS patients and 53 healthy controls were recruited from February 2019 to July 2020 from the First Affiliated Hospital of Chengdu Medical College. Eligible AIS participants were those: 1) diagnosed with AIS within 24 h onset, 2) aged between18 to 80 years, and 3) had provided signed informed consent. The study was approved by the ethics committee of Chengdu Medical College. None of the participants had received any anti-platelet and anti-coagulation treatment before obtaining blood samples. The major exclusion criteria were stroke, myocardial infarction, or head trauma in the last 3 months, and major surgery in the last 14 days. Healthy controls were those who had a physical examination during the same period. The clinical characteristics of the participants are shown in Table 1.

**TABLE 1:** Demographic and clinical characteristics of healthy control subjects and AIS samples.

Characteristics	HCs	AIS	*p* value
**Demographic characteristics**			
Total, n	53	53	
Age (mean ± SD), year	64.26 ± 10.04	67.92 ± 11.91	0.032*
Male, n (%)	17 (32.1)	30 (56.6)	0.011*
Asian race, n (%)	53 (100)	53 (100)	1.000
**Vascular risk factors**			
Pulse (beats/min)	77.45 ± 12.75	80 ± 12.72	0.306
Prior stroke history, n (%)	3 (5.7)	3 (5.7)	1.000
Smoking history, n (%)	10 (18.9)	16 (30.2)	0.223
Alcohol history, n (%)	3 (5.7)	5 (9.4)	0.470
Diabetes mellitus, n (%)	9 (17.0)	16 (30.2)	0.109
Hypertension, n (%)	26 (49.1)	30 (56.6)	0.436
Coronary disease, n (%)	1 (1.9)	6 (11.3)	0.051
**Laboratory parameters (mean ± SD)**			
Platelets, G/L	182.62 ± 58.53	180.7 ± 76.59	0.469
FIB (g/L)	3.98 ± 0.92	3.86 ± 0.92	0.500
PT (s)	11.36 ± 0.89	11.37 ± 0.89	0.805
APTT (s)	29.11 ± 2.92	29.8 ± 4.16	0.7
TT (s)	14.86 ± 1.07	14.97 ± 2.24	0.465
LDL (mmol/L)	2.59 ± 0.78	2.84 ± 1.21	0.413
HDL (mmol/L)	1.32 ± 0.32	1.38 ± 0.40	0.867
Total cholesterol (mmol/L)	4.33 ± 0.91	4.62 ± 1.30	0.385
Triglycerides (mmol/L)	1.56 ± 0.92	1.58 ± 1.49	0.167

HCs, healthy control subjects; AIS, acute ischemic stroke; FIB, fibrinogen; PT, prothrombin time; APTT, activated partial thromboplastin time; TT, thrombin time; LDL, low-density lipoprotein; HDL, high-density lipoprotein, *p < 0.05.

### Blood Collection and Platelet Isolation

5 ml whole blood from healthy donors and AIS patients were collected and added to ethylene diamine tetraacetic acid (EDTA) anti-coagulant. To avoid platelet activation during purification, we obtained plasma-free platelet suspensions according to the instruction manual of platelet purification kit (Shanghai Haling Bio, Shanghai, China) and stored at −80°C until use.

### RNA-Seq

We collected peripheral blood samples of five AIS patients and five healthy controls for MV preparations. The MV from serum was purified using Ribo™ Exosome Isolation Reagent (C10110-2, RIBOBIO, Guangzhou, China) and analyzed by flow cytometry and nanoparticle tracking analysis (NTA). The RNA from serum MV were extracted for quality inspection using Keo K5500/Qubit 2.0 and Agilent 2200 TapeStation. The cDNA library was constructed and sequenced with llumina HiSeq™ 2500 according to the manufacturer’s instructions.

### Bioinformatics Analysis

The target genes of differentially expressed miRNAs were analyzed using three types of miRNA target predictive databases, including miRTarBase (https://mirtarbase.cuhk.edu.cn/), RNA Interactome Database (RNAinter, http://www.rnainter.org/) and Encyclopedia of RNA Interactomes (ENCOR1, http://starbase.sysu.edu.cn/). To determine the potential biological functions and pathways, Gene ontology (GO) and Kyoto encyclopedia of genes and genomes (KEGG) pathway enrichment analysis were performed using the online software Metascape (https://metascape.org/).

### Analysis of Serum ET-1 and VEGFA Levels

We quantified serum levels of VEGFA and ET-1 in 53 AIS and 53 healthy donors by enzyme linked immunosorbent assay (ELISA) with VEGFA ELISA kit (MeiMian, Shanghai, China) and ET-1 ELISA kit (MeiMian, Shanghai, China).

### Reagents and Constructs

Chemical miR-200a-3p inhibitor, Cy5-miR-200a-3p mimic, miR-200a-3p mimic and their corresponding controls were all purchased from RiboBio Co., Ltd. (Guangzhou, China). The 3′UTR of MAPK14 wild-type (PGL3-MAPK14-wt), as well as mutant MAPK14 3′UTR (PGL3-MAPK14-mut) were cloned downstream of the firefly luciferase gene in the PGL3 plasmid. The following antibodies and reagents were also obtained: p38 MAPK antibody (p38, AF6456), phospho-p38 MAPK (p-p38, Thr180/Tyr182) antibody (AF4001), c-Jun antibody (AF6090), phospho-c-Jun (p-c-Jun, Ser73) antibody (AF3095), END1 (ET-1) antibody (DF6125), VEGFA antibody (AF5131), GAPDH antibody (AF7021) and horseradish peroxidase (HRP)-conjugated secondary antibody (S0001) were purchased from Affinity Biosciences (Suzhou, China). Anti-CD9 (380441), anti-CD63 (340219) and anti-TSG101 (381538) antibodies were obtained from ZEN-BIOSCIENCE (Chengdu, China).

### Cells and Transfection

HUVEC and 293T cells were obtained from China Cell Culture Center (Shanghai, China), cultured in Dulbecco’s Modified Eagle’s medium (DMEM) containing 10% fetal bovine serum (Gibco, Grand Island, NY, United States) and 100 U/ml penicillin-streptomycin. Lipofectamine 2000 reagent (Invitrogen, Carlsbad, CA, United States) was used for cell transfection. HUVEC were transfected with 80 nM miRNA mimic or inhibitor. Each transfection was performed at least three times.

### Luciferase Reporter Assays

We co-transfected pGL-MAPK14-wt or pGL-MAPK14-mut, pRL-CMV plasmids and miR-200a-3p mimic or mimic control into 293T cells. After 48 h of transfection, cells were harvested and detected using the dual luciferase report assay kit (Promega, Madison, WI, United States).

### Platelets Preparation, Transfection and PMVs Isolation

To generate miR-200a-3p-enriched PMVs, we collected 60 ml fresh whole blood from healthy controls added to EDTA anti-coagulant. Then, plasma-rich platelets (PRP) were obtained by centrifugation at 150 g for 10 min at room temperature (RT) twice to remove residual leukocytes and erythrocytes. Next, platelets were collected by centrifugation at 2000 g for 15 min, washed once using 6 ml 1 × HEPES (Macklin, Shanghai, China) and resuspended at 3 × 10^8^/ml in DMEM. The obtained platelets were transfected with 130 nM Cy5-miR-200a-3p mimic or negative control, and shaking cultured in 8 ml DMEM medium containing 1 IU/ml thrombin (Sigma) at 37°C, 120 rpm for 24 h. The PMVs were collected by centrifugation at 2000 g for 15 min, followed by centrifugation at 120,000 g for 2 h at 4°C with TL-100 ultracentrifuge (Beckman Coulter). The PMVs pellet was resuspended in 300 μL PBS, separated and stored at −80°C for use.

### PMVs Characterization and Incubation With HUVEC

We used nanoparticle tracking analysis (NTA; Malvern Instruments, Malvern, United Kingdom) and transmission electron microscopy (TEM; JEM-1220, Jeol, Tokyo, Japan) to assess the size and morphology of harvested PMVs. Next, we analyzed MV markers CD9, CD63 and TSG101 expression using western blot analysis. For incubation of PMVs with HUVEC, HUVEC were digested and seeded on 12-well plate 12–18 h before adding 100 μg PMVs into each well. After 24 h of co-incubation, HUVEC were obtained for qRT-PCR and quantitative protein assay.

### Incubation of Platelets and PMVs With HUVEC and Fluorescence Assays

For assessment of Cy5-miR-200a-3p transfer, HUVEC were seeded on coverslips 12–18 h before co-culture using a trans-well plate (0.4 μm polycarbonate filter, Corning). After transfection, approximately 1 × 10^8^ platelets were co-cultured with HUVEC in the presence or absence of 1 U/ml of thrombin. After 3, 6, 12 and 24 h co-culture, HUVEC were washed with PBS twice, fixed with 4% formaldehyde for 30 min, stained with nuclear dye hoechst 33258 (Solarbio, Beijing, China) for 5 min, and examined by fluorescence microscopy (Olympus corporation, Tokyo, Japan).

To analyze the adhesion of PMVs to HUVEC, PMVs were labeled with DiO-Membrane EVs labeling and Purification Kit (RENGEN BIOSCIENCES, Liaoning, China) according to the manufacture’s instruction, and co-incubated with HUVEC for 24 h. HUVEC were washed, fixed, stained with hoechst 33258 and examined by fluorescence microscopy.

### Quantitative RT-PCR

RNA of platelets was extracted using Trizol reagent (Invitrogen), quantified using a NanoDrop spectrophotometer and treated with RNAase-free DNase I (Invitrogen) as recommended by the manufacturer. For miRNA expression analysis, 0.5 μg RNA was added a polyA tail using mi*DETECT* A Track™ miRNA qRT-PCR Starter kit (RiboBio, Guangzhou, China), and reverse transcribed with mi*DETECT* A Track™ Uni-RT primer. Specific primers and SYBR green mix used to detect miRNAs were purchased from RiboBio Co., Ltd. The U6 endogenous control was used for normalization. For mRNA expression analysis, 0.5 μg RNA was reverse transcribed using a Superscript RT reagent kit (Takara). Specific primers for real-time PCR were listed in Table 2. β-actin was used for internal standard of mRNA quantification. The cycling conditions of mRNA qRT-PCR were as follows: 95°C for 30 s, followed by 40 cycles of 95°C for 15 s and 60°C for 30 s. Melting curve analysis was used for primers specificity detection.

**TABLE 2:** Primer sequence.

Gene primers	Sequences (5′→3′)
MAPK14-F	GAG​CGT​TAC​CAG​AAC​CTG​TCT​C
MAPK14-R	AGT​AAC​CGC​AGT​TCT​CTG​TAG​GT
c-Jun-F	CCT​TGA​AAG​CTC​AGA​ACT​CGG​AG
c-Jun-R	TGC​TGC​GTT​AGC​ATG​AGT​TGG​C
Endothelin 1(ET-1)-F	CTA​CTT​CTG​CCA​CCT​GGA​CAT​C
Endothelin 1(ET-1)-R	TCA​CGG​TCT​GTT​GCC​TTT​GTG​G
VEGFA-F	TTG​CCT​TGC​TGC​TCT​ACC​TCC​A
VEGFA-R	GAT​GGC​AGT​AGC​TGC​GCT​GAT​A
β-actin-F	TGG​CAC​CCA​GCA​CAA​TGA​A
β-actin-R	CTA​AGT​CAT​AGT​CCG​CCT​AGA

### Western Blot Analysis

Equal amounts of cell or PMVs lysate protein were separated in a 12% SDS-polyacrylamide gel, probed with antibodies against p38, p-p38, c-Jun, p-c-Jun, ET-1, VEGFA, as well as anti-human β-actin antibody. For PMVs marker identification, western blot analysis was performed with antibodies against CD9, CD63 and TSG101. The protein bands were detected with an ECL detection kit, analyzed by Image Lab software (BioRad) and quantified with Quantity One System (BioRad).

### Statistical Analysis

The results are expressed as the mean ± SEM of at least three independent experiments. Each data point of the qRT-PCR analysis represents the average value of three repetitions. Differences between two groups were assessed using the T-test or Mann–Whitney test. One-way ANOVA followed by Tukey’s post-hoc analysis was used to compare the differences in expressions of miR-200a-3p and miR-30a-5p among patients with different TOAST classifications. We assessed the correlation between serum ET-1 and VEGFA levels using the Spearman’s rank correlation coefficient. We performed receiver operating characteristic (ROC) analysis to assess the predictive performance of biomarkers. Differences are considered statistically significant at *p* < 0.05, and SPSS 26.0 was used for all statistical analyses (Chicago, Illinois, United States).

## Results

### Circulating microRNA Expression Profile in the Serum MV of AIS Patients

**FIGURE 1:**
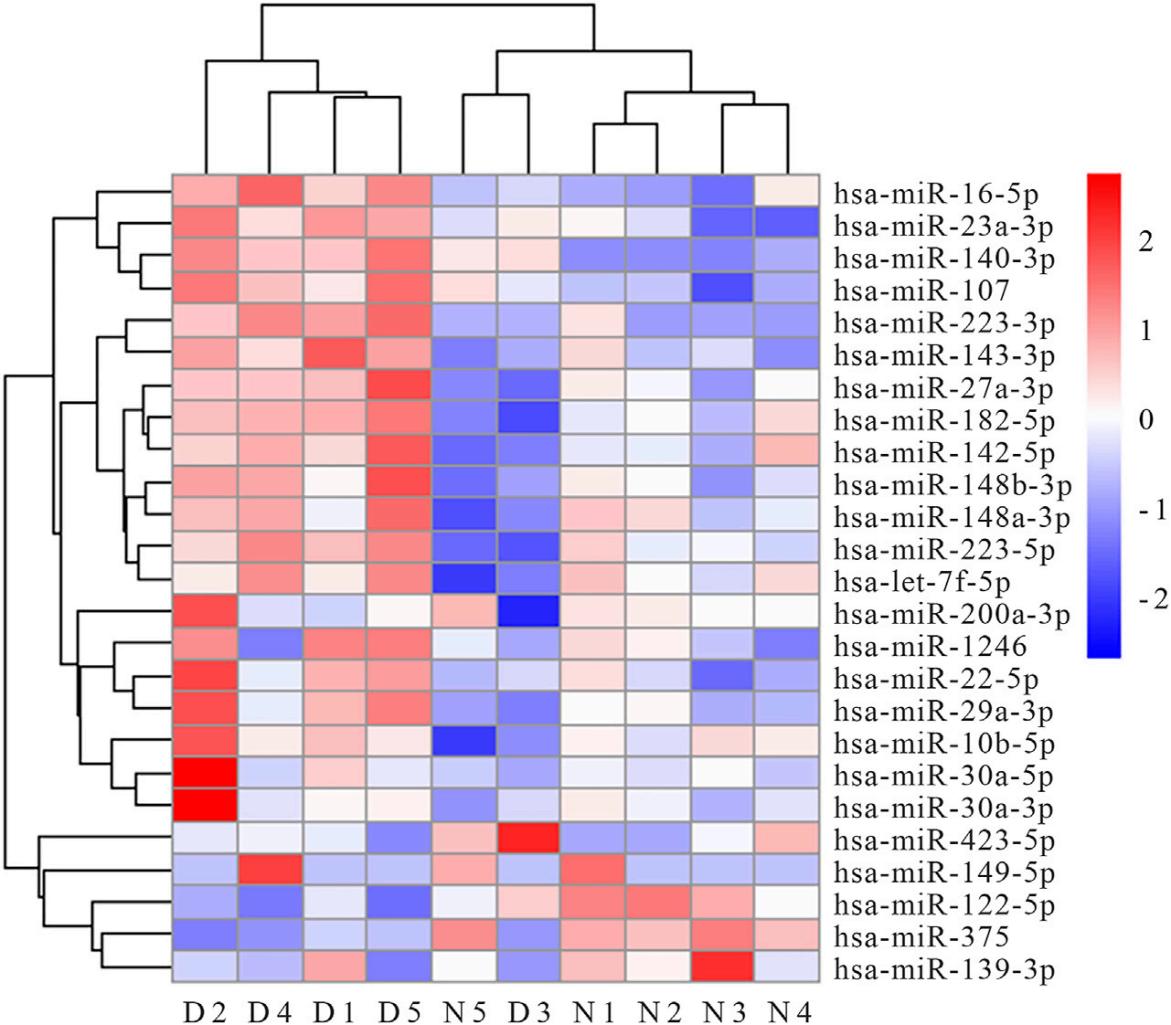
Heat map of top 25 miRNAs differentially expressed in serum MV of AIS patients and healthy controls (N1∼N5 represent healthy controls, D1∼D5 represent AIS patients).

We performed RNA-seq of serum MV in five AIS patients and five healthy controls, and 732 miRNA species were detected in total. Of note, the levels of 51 individual miRNA species were significantly different between AIS patients and healthy controls. The expression profiles of the top 25 miRNAs in serum MV are presented in the Figure 1 heat map. Five candidate miRNAs (miR-200a-3p, miR-30a-5p, miR-149-5p, miR-107 and miR-182-5p) were selected for further evaluation based on the expression abundance in serum, the differential expression profile, and the potential relevance to atherosclerosis, inflammation, or stroke.

### The Levels of Platelet miR-200a-3p and miR-30a-5p Were Up-Regulated in AIS

**FIGURE 2:**
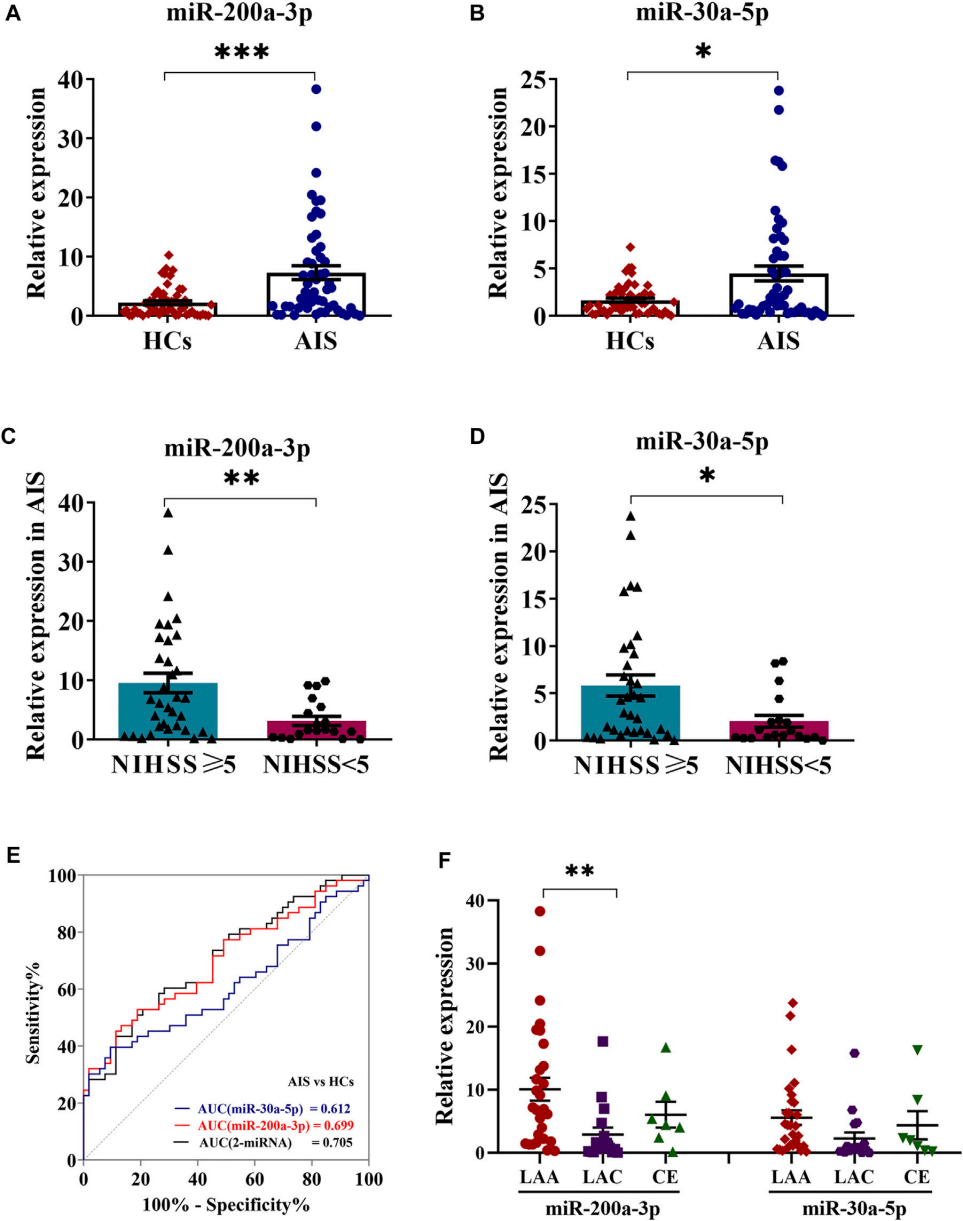
Expression levels of miR-200a-3p and miR-30a-5p in platelets. **(A,B)** QRT-PCR analysis was performed to detect the expression of platelet miR-200a-3p and miR-30a-5p in the AIS patients (*n* = 53) and HCs (*n* = 53). **(C,D)** The expression levels of platelet miR-200a-3p and miR-30a-5p in AIS patients with moderate to severe neurological deficit (NIHSS ≥ 5, *n* = 27) and mild neurological deficit (NIHSS < 5, n = 26). **(E)** Diagnostic value of a microRNA set for AIS. ROC curves for the individual miRNAs and the combination index and their corresponding AUC values for discriminating AIS patients (*n* = 53) from HCs (*n* = 53). **(F)** One-way ANOVA followed by Tukey’s post-hoc analysis was performed to compare miRNAs (miR-200a-3p and miR-30a-5p) differences in AIS patients by TOAST classification (LAA, *n* = 29; LAC, *n* = 7; CE, *n* = 17). Data were expressed as mean ± SEM. Differences between AIS patients and HCs were assessed by the Mann–Whitney U test, respectively, respectively, *p < 0.05, **p < 0.01, ***p < 0.001.

Considering platelets-derived MV constitute about two-thirds of total MV in peripheral blood, we next evaluated five miRNA expressions by qRT-PCR in platelets from 53 AIS patients and 53 healthy controls. Although the proportion of males with AIS was slightly higher than healthy controls, there were no statistical differences between the two groups in vascular risk factors and conventional laboratory parameters (Table 1). The qRT-PCR results demonstrated that levels of platelet miR-200a-3p (*p* < 0.001) and miR-30a-5p (*p* < 0.05) were significantly increased in AIS patients compared with the healthy controls (Figures 2A,B), whereas the levels of other three miRNAs were not markedly different (*p* > 0.05, Supplementary Figures S1A–C). Of these, the expressions of miR-200a-3p and miR-30a-5p were both elevated significantly in the platelets and serum MV of AIS patients. The miRNA expression levels of platelet miR-200a-3p and miR-30a-5p were significantly higher in AIS patients with severe neurological deficits (NIHSS≥5) than patients with mild neurological deficits (NIHSS < 5) (both *p* < 0.05, Figures 2C,D).

### The Predictive Value of Platelet miR-200a-3p and miR-30a-5p Expression Levels for the Diagnosis of AIS

Logistic regression analysis evaluating the association among age, gender and platelet miR-200a-3p and miR-30a-5p expression in AIS patients and healthy controls, indicated that platelet miR-200a-3p and miR-30a-5p were all likely to be independent risk factors for AIS. The ROC analyses for platelet miR-200a-3p and miR-30a-5p yielded area under the curve (AUC) of 0.699 and 0.612, cutoff values of 3.89 and 3.87, sensitivity of 53% and 40%, specificity of 81% and 91%, respectively. Interestingly, the AUC of the combination of platelet miR-200a-3p and miR-30a-5p was 0.705, with a sensitivity of 52% and a specificity of 81% at a cut-off value of 0.51 (Figure 2E). Further assessment of the differential expression of two miRNAs between AIS subtypes revealed that the expression of miR-200a-3p in platelets was significantly elevated in LAA patients compared with LAC (*p* < 0.01). However, platelet miR-30a-3p expression levels were similar among three subtypes (Figure 2F). Taken together, the results suggested that the combination of platelet miR-200a-3p and miR-30a-5p moderately predicts AIS occurrence.

### Functional and Pathway Enrichment Analysis of Two miRNAs

**FIGURE 3:**
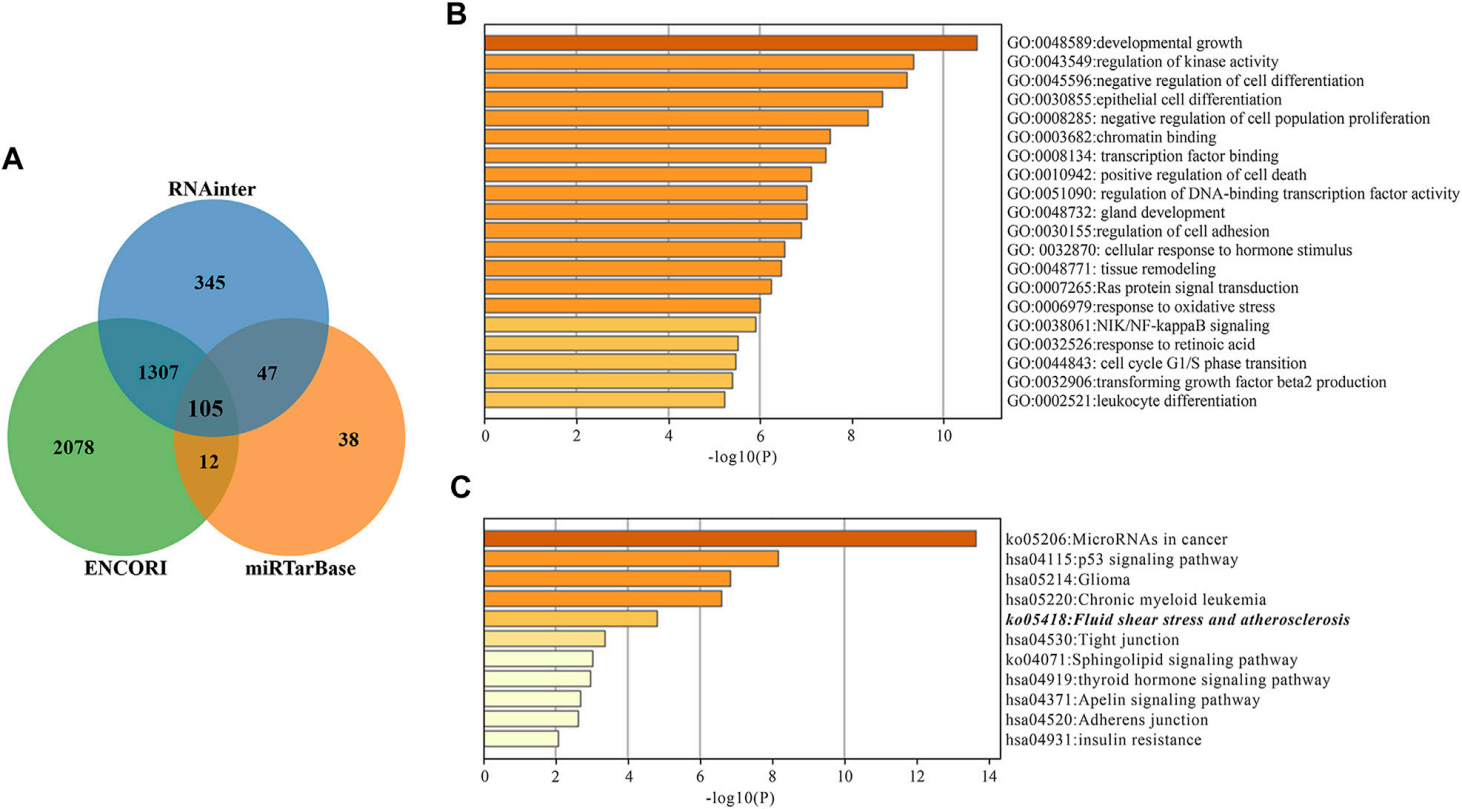
miR-200a-3p target gene prediction and enrichment analysis. **(A)** Wayne diagram of miR-200a-3p target genes predicted by miRTarBase, RNAinter and ENCORI databases. **(B)** Target genes GO Biological Processes of miR-200a-3p. **(C)** miR-200a-3p Target genes KEGG Pathway Analysis. The “Fluid shear stress and atherosclerosis” pathway is italicized.

Next, we explored the target mRNAs of two miRNAs and their roles in the AIS. For miR-200a-3p, 105 target genes were predicted simultaneously using three databases (miRTarBase, RNAinter and ENCOR1), as shown in Figure 3A. The potential functions of miR-200a-3p targets are focused on “developmental growth”, “regulation of kinase activity” and “negative regulation of cell differentiation” with GO analysis (Figure 3B). KEGG pathway enrichment analysis showed that miR-200a-3p target genes could be enriched to “Fluid shear stress and atherosclerosis” pathway (Figure 3C). Notably, there are six mRNAs that have predictive binding sites formiR-200a-3p in fluid shear stress and atherosclerosis pathways, including MAPK14, CTNNB1, PRKAA2, TP53, VCAM1 and KEAP1. We focused on MAPK14 (encoding p38) in this study, as it plays essential roles in inflammation and atherosclerosis. Given the results of the bioinformatics analysis (Supplementary Figures S2), the potential signaling pathways regulated by miR-30a-3p have little relevance to AIS pathophysiology, and so we have described it only briefly.

### Expression Levels of ET-1 and VEGFA Are Enhanced in the Serum of AIS Patients

**FIGURE 4:**
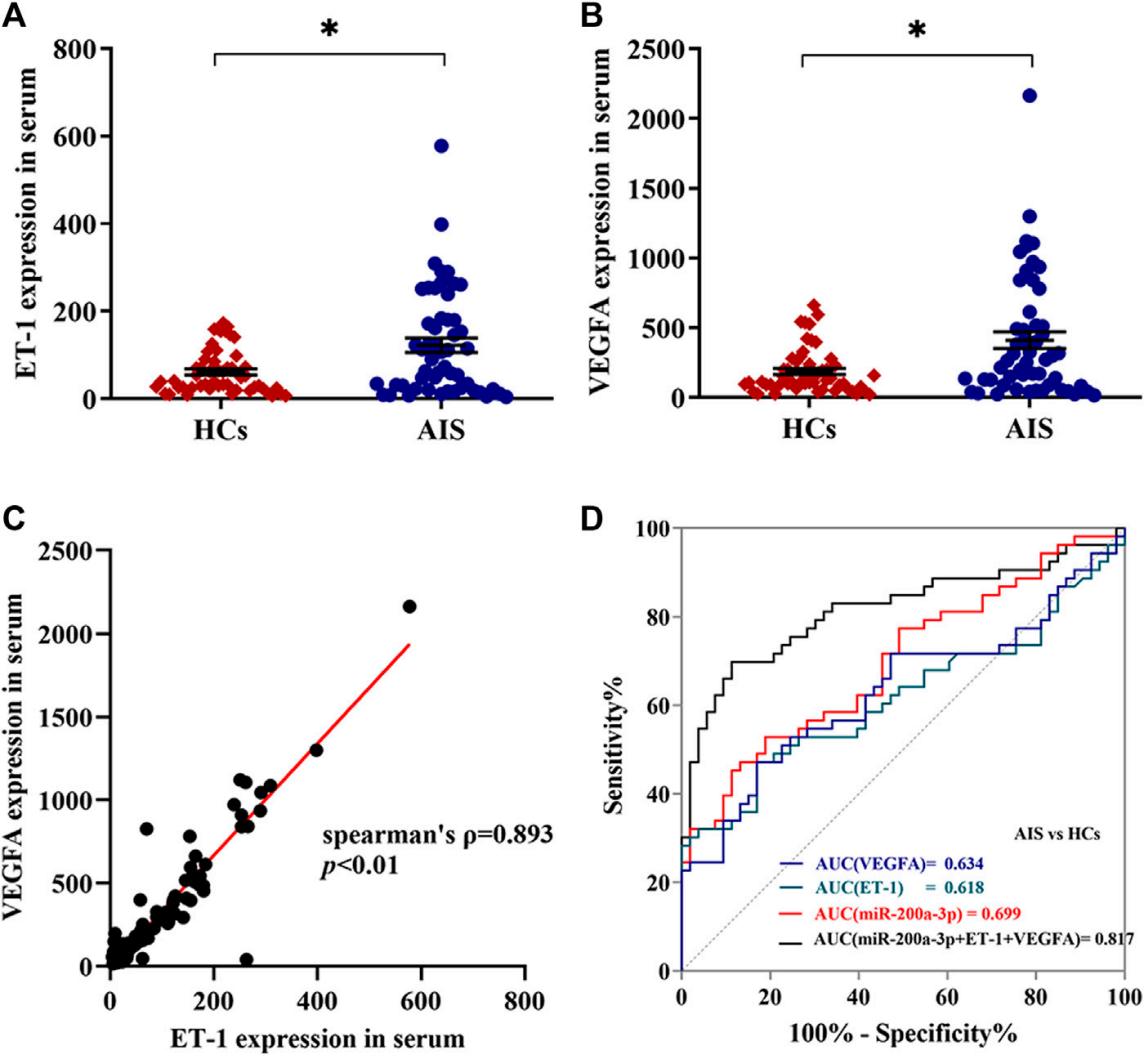
Expression levels of ET-1 and VEGFA in serum of AIS patients and healthy controls. **(A,B)** The expression levels of ET-1 and VEGFA in the serum of AIS patients and HCs were detected by ELISA. **(C)** The correlation between ET-1 levels and VEGFA levels in serum was assessed by the Spearman’s rank correlation coefficient, *n* = 106. **(D)** Diagnostic value of platelet miR-200a-3p, serum ET-1and VEGFA levels in AIS. ROC curves were generated based on serum ET-1 levels, VEGFA levels, and the combination index with platelet miR-200a-3p to distinguish AIS patients (*n* = 53) from normal controls (*n* = 53). Data were expressed as mean ± SEM. Differences between AIS patients and HCs were assessed by the Mann-Whitney U test, respectively, *p < 0.05.

Based on the bioinformatics analysis above, we hypothesized that platelet-derived miR-200a-3p may affect the ET-1 and VEGFA expression. To test this idea, we measured serum ET-1 and VEGFA levels by ELISA in the 106 participants. Compared with the healthy controls, the expression levels of ET-1 (124.35 ± 16.74 pg/ml versus 61.43 ± 6.81 pg/ml, *p* < 0.05) and VEGFA (416.04 ± 60.59 pg/ml versus 199.68 ± 25.16 pg/ml, *p* < 0.05) were markedly elevated in AIS patients (Figures 4A, B). Furthermore, we observed a positive correlation between serum ET-1 and VEGFA levels (Spearman ρ = 0.893, *p* < 0.01) (Figure 4C). The ROC curves for serum ET-1 and VEGFA showed the AUC was 0.618 and 0.634; the cutoff values were 111.36 pg/ml and 295.70 pg/ml, the sensitivity values were both 47% and the specificity values were 83% and 81%, respectively. In addition, the combination of serum ET-1, VEGFA, and platelet miR-200a-3p had an AUC of 0.817, a sensitivity of 70%, and a specificity of 89% using a predictive risk algorithm (Figure 4D). These results indicated that the combination of serum ET-1, VEGFA and platelet miR-200a-3p moderately predicts AIS occurrence.

### miR-200a-3p Inhibits p38 MAPK Signaling Pathway *via* Targeting MAPK14

**FIGURE 5:**
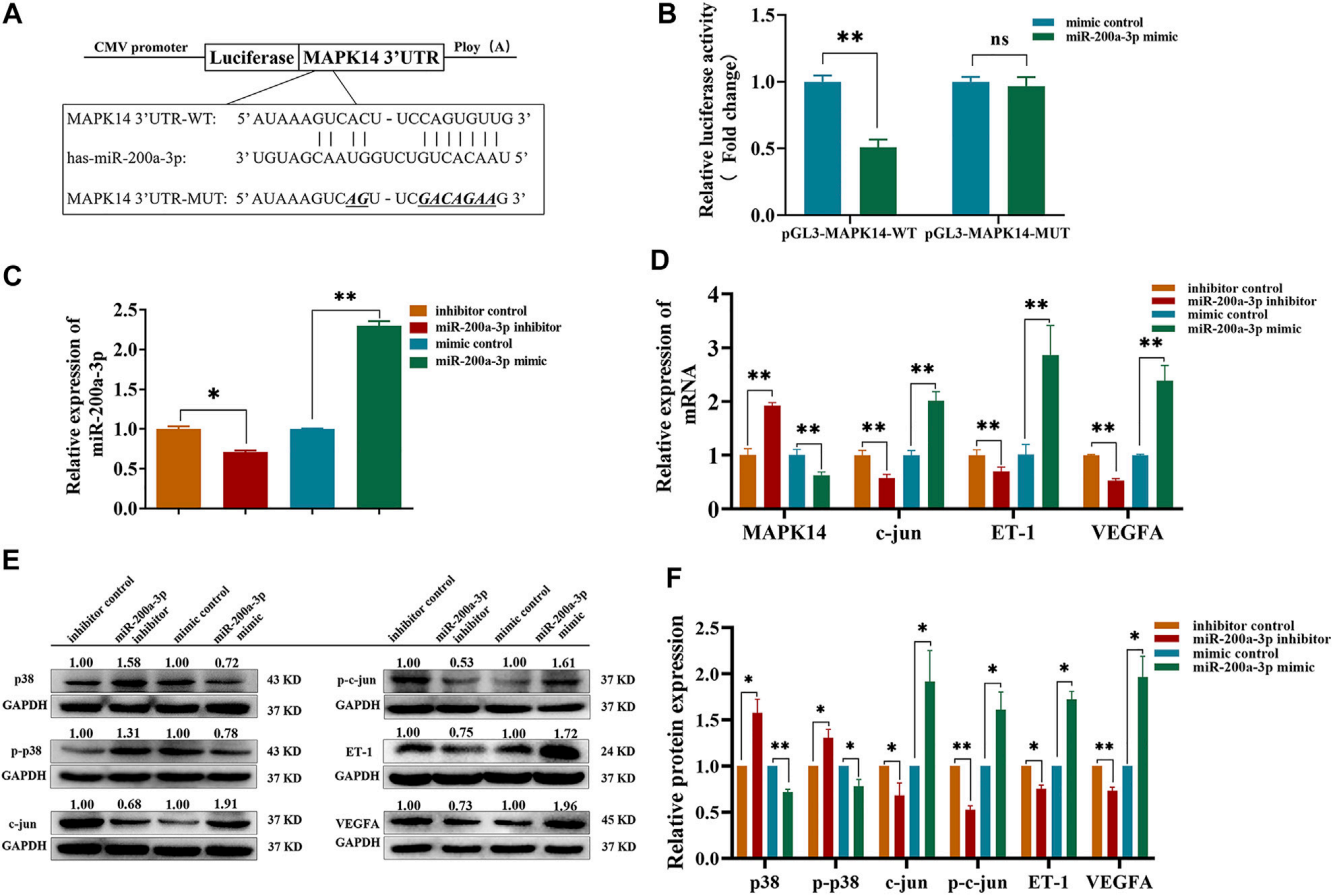
miR-200a-3p directly targets the 3′UTR of MAPK14 and regulates the expression levels of p38, c-jun, ET-1 and VEGFA. **(A)** The MAPK14 3′-UTR containing the wildtype or mutant miR-200a-3p binding sequence was inserted into downstream of the luciferase reporter vector. The mutated sequences are italicized. **(B)** The dual luciferase reporter assay revealed that the luciferase activity controlled by MAPK14 3′-UTR was inhibited by ectopic miR-200a-3p expression in 293T cells. **(C)** miR-200a-3p was highly expressed or knocked down in HUVEC by lipofectamine 2000 transfection. QRT-PCR analysis was performed to measure the expression levels of miR-200a-3p in HUVEC after treatment with miR-200a-3p mimic, mimic control or miR-200a-3p inhibitor, inhibitor control. **(D)** The mRNA levels of MAPK14, c-jun, ET-1 and VEGFA were determined by qRT-PCR in HUVEC transfected with miR-200a-3p mimic, mimic control or miR-200a-3p inhibitor, inhibitor control. **(E)** Western blot analysis of p38, phosphorylated p38, c-jun, phosphorylated c-Jun, ET-1 and VEGFA protein levels in HUVEC transfected with miR-200a-3p mimic, mimic control or miR-200a-3p inhibitor, inhibitor control. **(F)** Quantification of protein results in panel E. Data were expressed as mean ± SEM. Between group differences were assessed by the student’s t test, respectively. *p < 0.05, **p < 0.01, ns, not significant.

Though MAPK14 has been verified as a target of miR-200a-3p in liver cells before (Xiao et al., 2015), its modulation in endothelial cells requires further investigation. We cloned the targeting region of MAPK14 3′UTR into a luciferase report plasmid (Figure 5A). The report plasmids were co-transfected into 293T cells along with miR-200a-3p mimic. The dual luciferase reporter assay illustrated that miR-200a-3p significantly down-regulated the luciferase activity with the wild-type but not the mutated MAPK14 3′UTR (Figure 5B). Both gain-of-function and loss-of-function analysis in HUVEC also demonstrated that the suppression of miR-200a-3p on the expression of endogenous MAPK14 mRNA and protein levels. QRT-PCR analysis showed that miR-200a-3p mimic transfected HUVEC led to a significant 2.30 times increase in miR-200a-3p expression, 1.59 times decrease in MAPK14 mRNA, 2.01 times increase in c-jun mRNA, 2.87 times increase in ET-1 mRNA and 2.39 times increase in VEGFA mRNA. On the contrary, the depletion of miR-200a-3p in HUVEC transfected with miR-200a-3p inhibitor led to a 1.43 times decrease in miR-200a-3p expression, 1.92 times increase in MAPK14 mRNA, 1.75 times decrease in c-jun mRNA, 1.43 times decrease in ET-1 mRNA and a 1.89 times decrease in VEGFA mRNA levels, respectively (Figures 5C,D). Similar results were yielded using western blot analysis. miR-200a-3p mimic transfection showed 1.39 and 1.28 times decrease in the total p38 protein and phosphorylated p38 protein, 1.91 and 1.61 times increase in total c-jun protein and phosphorylated c-jun protein, 1.72 and 1.96 times increase in ET-1 protein and VEGFA protein. Accordingly, miR-200a-3p inhibition led to an approximately 1.58 and 1.31 times increase in total p38 protein and phosphorylated p38 protein, 1.47 and 1.89 times decrease in total c-jun protein and phosphorylated c-jun protein, a 1.33 times decrease in ET-1 protein and a 1.37 times decrease in VEGFA protein, respectively (Figures 5E,F). These results suggested that miR-200a-3p may enhance ET-1 and VEGFA expression by suppressing p38 MAPK signaling pathway in endothelial cells *via* targeting MAPK14.

### miR-200a-3p is Transferred From Platelets to Endothelial Cells

**FIGURE 6:**
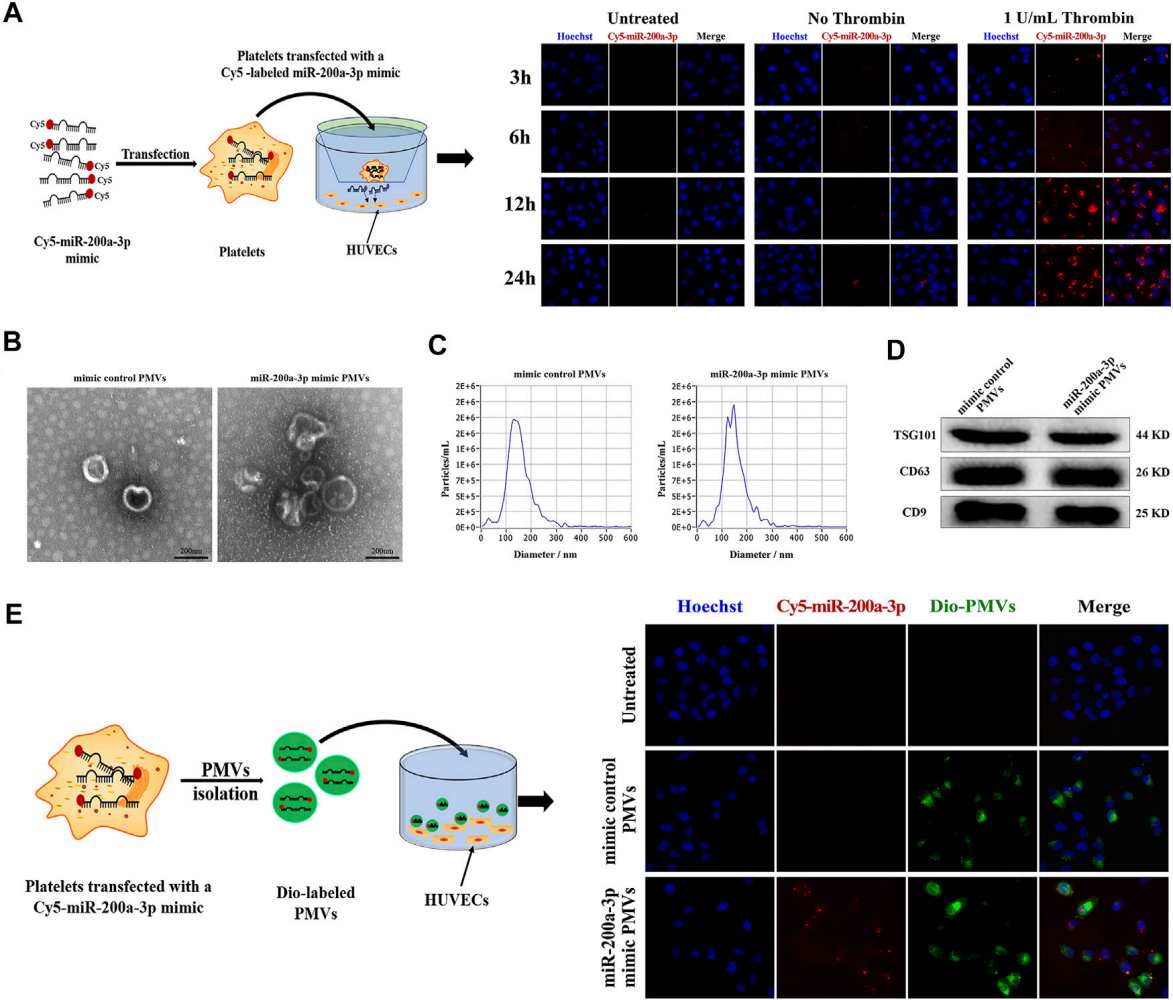
Effective delivery of platelet miR-200a-3p into HUVEC by PMVs. **(A)** Representative fluorescence images of HUVEC after 3, 6, 12 and 24 h co-culture with Cy5-labeled miR-200a-3p mimics (red) transfected platelets in the presence or absence of 1 U/mL thrombin at 37°C. Nuclei were stained blue. **(B)** Representative transmission electron microscopy images of PMVs isolated using ultracentrifugation (scale bar, 200 nm). **(C)** The size distribution and concentration of isolated PMVs measured by Nanoparticle Tracking Analysis. **(D)** The PMVs specific markers TSG101 and CD63, and CD9 were detected by western blot analysis. **(E)** After platelets were transfected with mimic control or Cy5-labeled miR-200a-3p mimic (red), PMVs were isolated by ultracentrifugation. Representative fluorescence images of HUVEC incubated with DiO green fluorescence-labeled PMVs in the presence of 1 U/mL thrombin for 24 h at 37°C. Nuclei were stained blue.

In the presence of thrombin-activated platelets, the red fluorescence intensity in co-cultured HUVEC was enhanced at 12 h and peaked at 24 h (Figure 6A). In contrast, the red fluorescence intensity in HUVEC co-cultured without platelets (negative control) or with resting platelets (without thrombin treatment) was mostly negligible at the indicated time, showing that the release of Cy5-miR-200a-3p from activated platelets was enhanced. These results indicated that Cy5-miR-200a-3p was effectively delivered from activated platelets into HUVEC.

### Delivery of miR-200a-3p by PMVs Into HUVEC *in vitro*

Given the recent reports, miRNA-containing PMVs released from activated platelets alter recipient cell function through miRNA delivery (Liang et al., 2015; Badimon et al., 2016; Shu et al., 2019). We obtained miR-200a-3p-enriched PMVs by transfection of platelets with fluorescent Cy5-labeled miR-200a-3p mimic, followed by stimulation with 1 U/mL thrombin for 24 h. PMVs was characterized using TEM, NTA and western blot analysis. Most nanoparticles exhibited clear cup-shaped or spherical morphology using TEM analysis (Figure 6B). Then, we directly tracked the size of PMVs with the dynamic light scattering analysis system NTA and found that the average particle size was about 150 nm, which was consistent with the diameter of most PMVs concentrated at 30–300 nm (Figure 6C). We further confirmed the surface markers CD9, CD63 and TSG101 expression of PMVs by western blot analysis (Figure 6D), indicating that PMVs were successfully isolated from activated platelets. Furthermore, to confirm that PMVs could deliver miR-200a-3p to endothelial cells, the PMVs were labeled with the green fluorescent dye DiO and then co-cultured with HUVEC. After 24 h co-incubation, HUVEC exhibited efficient uptake of miR-200a-3p as indicated by the presence of green and red fluorescent staining in HUVEC (Figure 6E).

### Gene Expression in the MAPK Signaling Pathway is Altered by miR-200a-3p-Enriched PMVs in HUVEC

**FIGURE 7:**
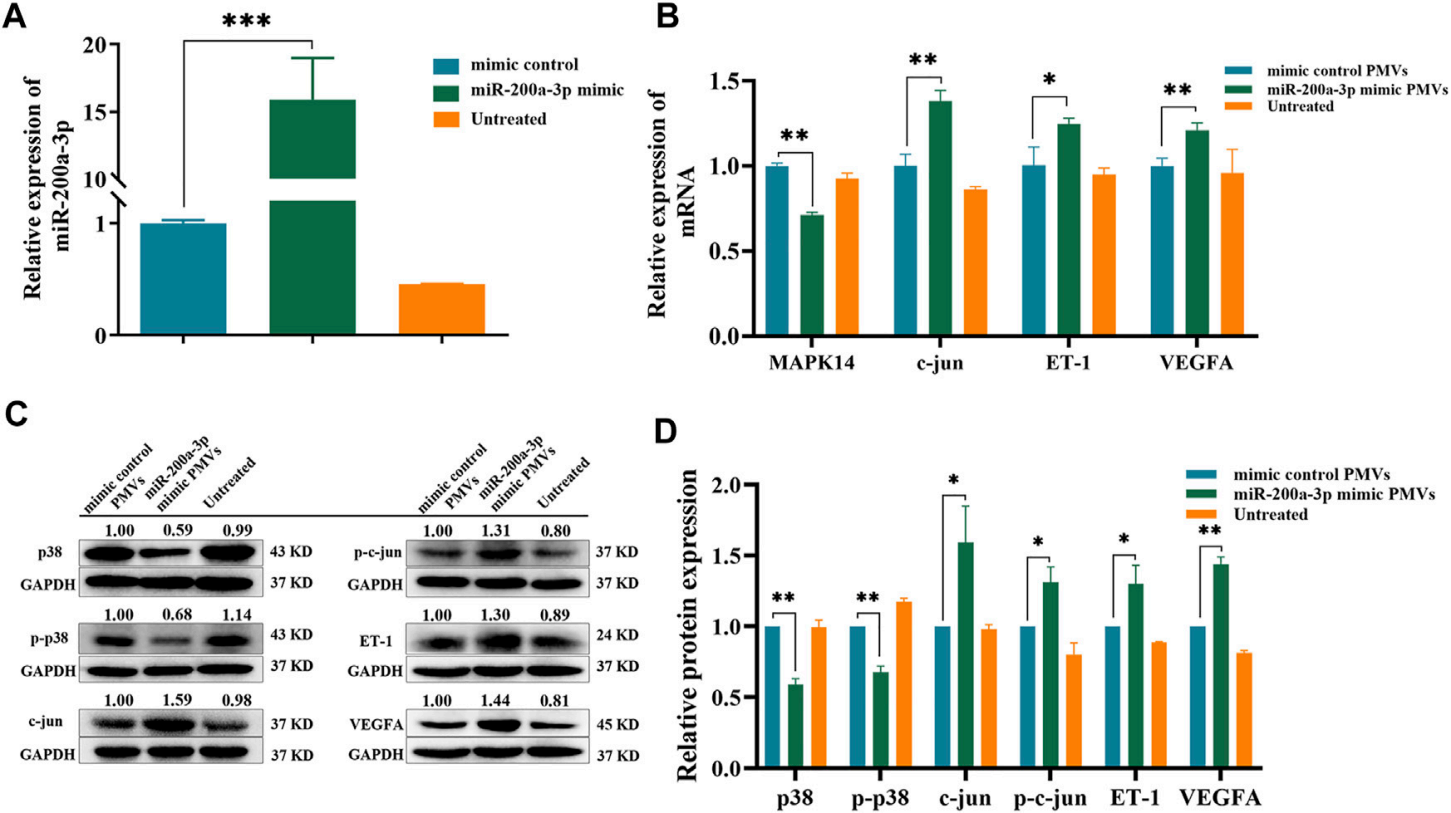
Regulation of p38/c-jun pathway by PMVs in HUVEC. **(A)** PMVs were isolated by ultracentrifugation after transfecting platelets with mimic control or miR-200a-3p mimic. QRT-PCR analysis of miR-200a-3p expression levels in HUVEC treated without PMVs (untreated) or with PMVs from various sources (mimic control PMVs and miR-200a-3p mimic PMVs) for 24 h. **(B)** MAPK14, c-jun, ET-1 and VEGFA mRNA levels in HUVEC after treatment with or without PMVs from various sources were determined by qRT-PCR. **(C)** Western blot analysis relative expression levels of p38, phosphorylated p38, c-jun, phosphorylated c-Jun, ET-1 and VEGFA protein in HUVEC treated with or without PMVs from various sources. **(D)** Quantification of protein results in panel C. Data were expressed as mean ± SEM. Differences between data were assessed by the student’s t test, *p < 0.05, **p < 0.01, ***p < 0.001.

Finally, to confirm whether miR-200a-3p-enriched PMVs could regulate the target gene expression in the endothelial cells, we co-cultured PMVs and HUVEC for 24 h. As shown in Figure 7A, compared with the control PMVs treatment, the levels of miR-200a-3p were significantly raised in HUVEC exposed to miR-200a-3p-enriched PMVs, indicating that PMVs could effectively deliver miR-200a-3p into HUVEC. Moreover, the mRNA levels of MAPK14 were down-regulated by 1.43 folds (*p* < 0.05), whereas the mRNA levels of c-Jun, ET-1 and VEFGA significantly increased by 1.38, 1.23 and 1.22 folds, respectively (Figure 7B). As expected, western blot analysis showed that the protein levels of total p38 and phosphorylated p38 decreased by 1.69 and 1.47 folds, while the protein levels of c-jun, phosphorylated c-jun, ET-1 and VEGFA increased by 1.59, 1.31, 1.30 and 1.44 folds in HUVEC co-incubated with miR-200a-3p-enriched PMVs compared with control PMVs (Figures 7C,D). Collectively, these data showed the functional role of miR-200a-3p-enriched PMVs on ET-1 and VEGFA expression in endothelial cells, consistent with the results of overexpression and knockdown of miR-200a-3p in HUVEC (Figure 5).

## Discussion

The management of AIS worldwide is currently limited by our incomplete understanding of the cellular and molecular mechanism, lacking early diagnosis biomarkers, and having a narrow therapeutic window (Mendelson and Prabhakaran, 2021). Thus, understanding of the underlying mechanisms of AIS occurrence will help with the development of novel diagnostic and therapeutic targets for AIS patients. In recent years, circulating microvesicles containing miRNAs have emerged as early diagnostic markers for various diseases, prognostic stratification, and identification of potential therapeutic targets (Femminò et al., 2020; Mohan et al., 2020). To our knowledge, the miRNA transcriptome of serum microvesicles in AIS patients has not yet been reported.

We studied miRNA expression profiles in the serum MV of AIS patients and healthy controls using RNA-seq. Unlike microarray as a hybridization-based technique, the results of RNA-seq are thought to be more unbiased (van der Brug et al., 2010). Consistent with the results of RNA-seq, the expression levels of miR-200a-3p and miR-30a-5p were systemically up-regulated in platelets of AIS patients, which prompted us to consider whether the platelets activation after AIS leads to a release of miRNA from platelets.

Anucleate platelets contain a high ratio of pre-miRNA/miRNA and the enzymes, which are required to transform the pre-miRNA into mature miRNA (Pan et al., 2014; Rowley et al., 2016). Platelets contain at least 170 different miRNA species, that are widely involved in many processes, especially inflammation, platelet activation, endothelial functions, and pathophysiology of cardiovascular diseases (Fejes et al., 2018; Khodadi, 2020). These miRNAs in platelets mainly mediate the post-transcriptional regulation of gene expression. Some of the miRNAs released from activated platelets are packaged by microvesicles, that may remotely mediate cell-to-cell communication. miRNA-containing MV released from activated platelets can enter endothelial cells and regulate ICAM1 expression by blocking NF-κB and the MAPK pathways, thus platelet MV plays a major role in inflammation and atherosclerosis (Gidlöf et al., 2013).

Our present results confirm the importance of p38 MAPK/JNK signal transduction pathways in inducible ET-1 and VEGFA expression. The overexpression of miR-200a-3p downregulated total p38 and phosphorylated p38 protein levels, and significantly enhanced c-Jun gene expression. The relative gene expression is further verified with the knockdown of miR-200a-3p in HUVEC. As well know, transcription factor c-Jun is activated *via* phosphorylation by JNK MAPK (Dobreva et al., 2009). The results showing that the inhibition of p38 activity may lead to a compensatory upregulation of c-Jun gene expression in HUVEC, are consistent with findings of Kayahara et al., which indicated activated p38 negatively affects c-Jun expression in an EGF-dependent manner (Kayahara et al., 2005).

Furthermore, we showed that the serum expression levels of ET-1 and VEGFA are strongly and positively correlated and were significantly elevated in AIS patients (Figures 4A–C), which is consistent with previous literature (Giannopoulos et al., 2008; Bhasin et al., 2019). ET-1 (the major isoform of endothelins) and VEGFA, which have been both reported to play a key role in the pathogenesis of atherosclerosis, cardiovascular disease and stroke, may provide potential novel therapeutic targets (Shibuya, 2015; Jung et al., 2016; Barton and Yanagisawa, 2019). However, the underlying mechanism of up-regulation of ET-1 and VEGFA in serum is still unclear. For the first time, we revealed that the expression levels of ET-1 and VEGFA were markedly promoted by miR-200a-3p *via* targeting MAPK14 in HUVEC, which sheds new light on the underlying mechanism of elevated expression of ET-1 and VEGFA in AIS patients. Interestingly, we found that the combination of platelet miR-200a-3p levels with serum ET-1 and VEGFA levels could be a strong predictive biomarker for AIS occurrence (Figure 4D).

The current study has two limitations. Firstly, we have a relatively small sample of AIS patients recruited from a single center. Secondly, the regulatory function of the miR-200a-3p-containing PMVs on endothelial cell function would need to be investigated *in vivo*.

Next, we will further study the effect of miR-200a-3p on endothelial cell function, such as endothelial cell proliferation, apoptosis, angiogenesis, etc. The effect of miR-200a-3p knockdown on endothelial cell function *in vivo* will be investigated on a cerebral ischemia mouse model. Furthermore, we are also interested in the pre-miRNA expression profiles in platelets of AIS patients to explore the reasons for the change of miRNA expression in platelets.

In summary, we discovered that activated platelets could remotely modulate ET-1 and VEGFA levels in vascular endothelial cells through the release of miR-200a-3p-containing PMVs *via* targeting MAPK14, which provides a potential miRNA-based therapeutic strategy for atherosclerosis related diseases. Moreover, our results suggested that miR-200a-3p levels in platelets were positively associated with the risk of AIS, and the combination of platelet miR-200a-3p and serum ET-1 and VEGFA levels significantly improved the prediction of AIS.

## Data Availability

The original contributions presented in the study are publicly available. This data can be found here: https://www.ncbi.nlm.nih.gov/bioproject/, PRJNA822305; GEO: GSE199942.

## Abbreviations

AIS, acute ischemic stroke; MV, microvesicles; PMVs, platelets-derived microvesicles; 3′UTR, 3′ untranslated region; ET-1, endothelins 1; VEGFA, vascular endothelial growth factor A; PRP, platelet-rich plasma; NTA, nanoparticle tracking Analysis; TEM, Transmission electron microscopy; QRT-PCR, quantitative RT-PCR; ELISA, enzyme linked immunosorbent assay; NIHSS, national institute of health stroke scale; AUC, area under the curve; LAA, large artery atherosclerosis; CE, cardioembolism; LAC, lacunar infarct; ODE, stroke of other determined etiology; UDE, stroke of undetermined etiology.
