# Supplementary material for: Platelets-Derived miR-200a-3p Modulate the Expression of ET-1 and VEGFA in Endothelial Cells by Targeting MAPK14
Source: Front Physiol. 2022 Jun 9;13:893102. doi: 10.3389/fphys.2022.893102 (PMC9224407; doi:10.3389/fphys.2022.893102)
Supplement: Supplementary file 1 [file DataSheet1.DOCX]

Supplementary Material

# Supplementary Figures


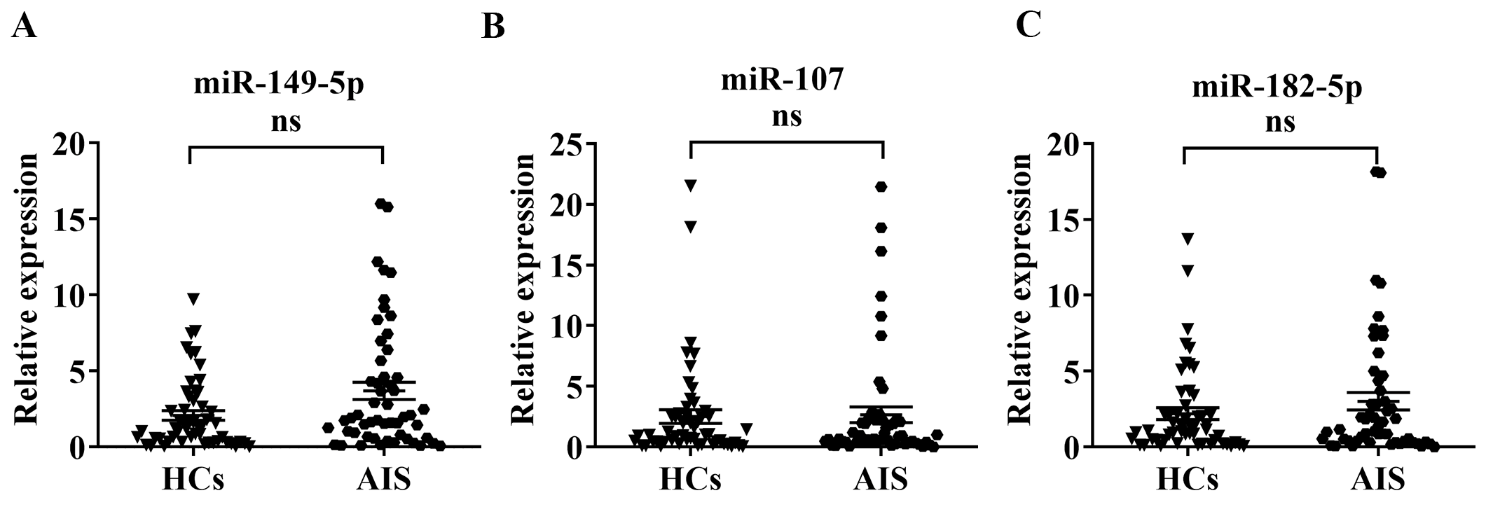


**Supplementary Figure 1.** Expression levels of platelet miRNAs.

(A-C) QRT-PCR analysis was performed to detect the expression of platelet miRNAs in both the AIS patients and healthy controls. n = 53 in stroke group and n = 53 in control group. Data were expressed as mean ± SEM. Differences of data in both the AIS patients and healthy controls were assessed by the Mann–Whitney U test, respectively, ns, not significant.


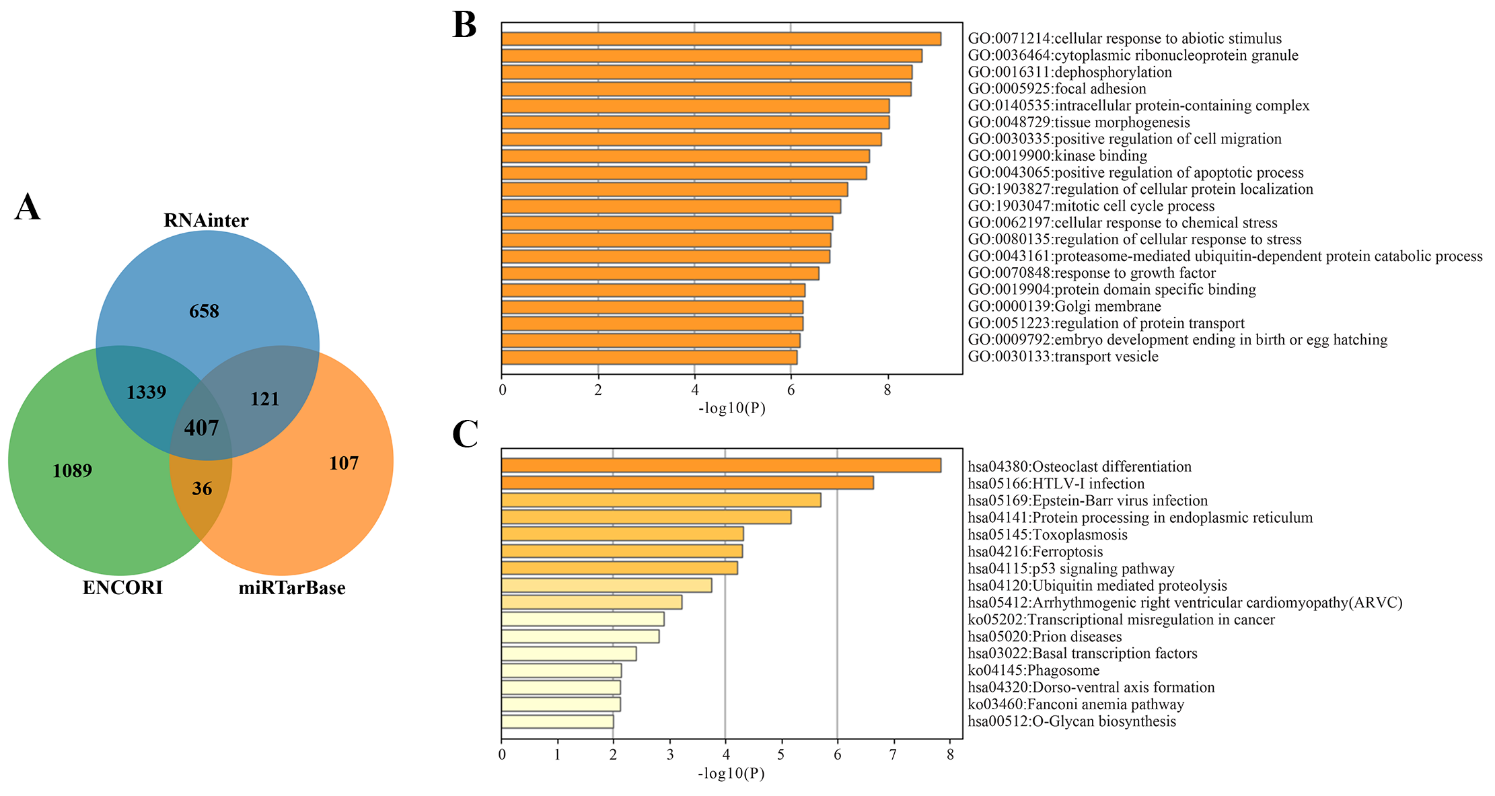


**Supplementary Figure 2.** miR-30a-5p target gene prediction and enrichment analysis.

(A) Wayne diagram of miR-30a-5p target genes predicted by miRTarBase, RNAinter and ENCORI databases.

(B) Target genes GO Biological Processes of miR-30a-5p.

(C) miR-30a-5p target genes KEGG Pathway Analysis.
